# From Triage to Outcome: How ED Parameters and Comorbidities Shape Pediatric Critical Illness Prognosis

**DOI:** 10.3390/children13091151

**Published:** 2026-08-27

**Authors:** Ian Valencic, Fiorentina Guida, Laura Andreozzi, Ilaria Corsini, Eleonora Battelli, Marcello Lanari, Daniele Zama

**Affiliations:** 1 Specialty School of Paediatrics—Alma Mater Studiorum, University of Bologna, 40138 Bologna, Italy; 2 Pediatric Unit, IRCCS Azienda Ospedaliero-Universitaria di Bologna, 40138 Bologna, Italy; fiorentina.guida@unibo.it (F.G.); laura.andreozzi4@unibo.it (L.A.); ilaria.corsini2@unibo.it (I.C.); eleonora.battelli@aosp.bo.it (E.B.); marcello.lanari@unibo.it (M.L.); daniele.zama2@unibo.it (D.Z.); 3 Department of Medical and Surgical Sciences, University of BolognaVia Giuseppe Massarenti, 9, 40138 Bologna, Italy

**Keywords:** pediatric emergency department (PED), advanced vital support (ALS), high-priority code, red code (RC), chronic health conditions (CCs)

## Abstract

**Highlights:**

**What are the main findings?**

- This study provides a portrait of the pediatric population presenting with recurrent crises (RC) that is broadly consistent with the international literature, while also reflecting the specific characteristics of the local healthcare system. Among patients presenting with the highest severity code, 56.21% had at least one pre-existing chronic condition. Neuropsychiatric conditions were the most frequent comorbidities, followed by cardiovascular and onco-hematological conditions. Among children with chronic conditions, 25% were followed by pediatric palliative care services, providing further insight into the complexity and healthcare needs of this population.
- Younger age was associated with hospital and PICU admission, whereas neither age nor clinical severity was significantly associated with outcomes assessed at 7 days. In our cohort, clinical severity, as reflected by the need for advanced life support in the PED rather than by an elevated clinical score, showed a stronger association with PICU admission. Despite the severity of some initial presentations, the small number of children requiring ongoing vital support at 7 days suggests an overall favorable short-term recovery.

**What are the implications of the main findings?**

- These findings highlight the need for structured follow-up of children with chronic conditions and for strengthening tertiary prevention strategies within the community care network. Greater continuity and coordination of care across healthcare settings may facilitate early identification and management of clinical needs, potentially reducing avoidable pediatric emergency department admissions in the coming years.
- The association between younger age and hospitalization may partly reflect a more cautious clinical approach when managing younger children. At the same time, deviations in clinical parameters in the ED should not be considered in isolation or as a substitute for a comprehensive assessment of the underlying condition. Although advanced life-support interventions were rarely required, their timely application in the early management of critically ill children remains essential for achieving favorable outcomes. These findings underscore the importance of maintaining and strengthening pediatric emergency competencies through targeted training and ongoing skills development.

**Abstract:**

**Objective:** To describe the characteristics of children presenting with red-code triage to a tertiary Pediatric Emergency Department (PED) and to evaluate the association between variables at PED presentation and hospitalization outcomes. **Methods**: This retrospective study included patients aged 0–16 years presenting to the PED with highest level of urgency at triage between 2021 and 2025. Demographic, clinical, laboratory, and outcome data were extracted from electronic medical records. Vital signs at presentation were categorized by severity using age-adjusted percentiles and established thresholds. Outcomes included hospital admission, PICU admission, need for advanced vital support (ALS) within 7 days, and hospitalization at 7 days. Univariable and multivariable logistic regression analyses were performed to identify predictors of outcomes. **Results:** Critically ill pediatric presentations accounted for 0.23% of PED attendances. Chronic comorbidities were present in 56.21%, mainly neuropsychiatric conditions. Seizures (33.99%) and respiratory conditions (25.49%) were the leading causes. Most patients were hospitalized (56.86% in ward, 20.26% in PICU). Invasive ventilation was required in 7.18% of patients and inotropic support in 3.92%. Increasing age was associated with significantly lower odds of both hospital admission (OR: 0.876, 95% CI: 0.783–0.976; *p* = 0.017) and PICU admission (OR: 0.890, 95% CI: 0.793–0.983; *p* = 0.031). Conversely, the need for advanced life support in the ED was independently associated with PICU admission (OR: 3.247, 95% CI: 1.345–7.997; *p* = 0.009). None of the evaluated ED clinical parameters were independently associated with 7-day outcomes. **Conclusions:** Our cohort demonstrates a high prevalence of chronic comorbidities, confirming their growing relevance in hospital resource allocation. Life-saving interventions were rarely required, underscoring the need for strategies to maintain medical competencies. Younger age was associated with an increased risk of hospitalization. Monitoring of clinical parameters in the ED, although a useful complementary measure, cannot replace a comprehensive medical assessment of the underlying condition.

## 1. Background

The pediatric emergency department (PED) is a cornerstone of healthcare systems and a key indicator of hospital care quality, with an estimated rate of 34 visits per 100 children. Patients are triaged using priority codes; the highest level indicates impairment of one or more vital functions requiring immediate assessment. High-priority cases account for 0.2% to 0.7% of visits, although the data remains limited. These cases represent a heterogeneous group that require diverse diagnostic and therapeutic pathways, and the scarcity of data may hinder staff preparedness and the development of targeted emergency care programs [1,2,3,4,5].

The literature requires periodic updating as relevant clinical and epidemiological variables evolve over time. These changes reflect both progressive trends, such as the increasing prevalence of comorbidities in the pediatric population, and disruptive events, including the SARS-CoV-2 pandemic, as well as the introduction of medical innovations (e.g., universal prophylaxis with nirsevimab) [6,7,8]. This variability in clinical approaches to pediatric emergencies is further driven by differences in healthcare systems, which are shaped by local context and result in distinct probabilities of receiving diagnostic workups, medical interventions, and even hospitalization; therefore, the study of clinical practice is essential to identify areas with potential for improvement in patient care [5,9].

The aim of this study was to describe the characteristics of children presenting with RC triage to a tertiary PED and to evaluate the association between clinical and demographic variables at PED presentation and hospitalization outcomes.

## 2. Methods

### 2.1. Emergency Department Organization

This monocentric, observational, retrospective cohort study at the PED of the IRCCS Azienda Ospedaliero-Universitaria in Bologna (Italy) Policlinico of Sant’Orsola, a tertiary-level hospital that manages approximately 24,000 visits per year, was approved by the ethics committee (No. 601/2025/Oss/AOUBo).

Sant’Orsola Hospital is a referral center for pediatric cardiologic, onco-hematologic, and neurologic diseases.

The hospital provides a pediatric intensive care unit (PICU), a neonatal intensive care unit, a cardiac intensive and intermediate care unit, and pediatric general and specialized wards, and a Short Observation Unit (SOU).

The triage system is based on a global, patient- and family-centered approach and relies on nurse-led assessment, including the collection of both subjective data (e.g., patient-reported symptoms and identification of the chief complaint) and objective data (e.g., measurement of vital signs). The model is structured into five priority codes based on risk stratification and the patient’s level of clinical severity. RC (priority level 1; Supplementary Table S1) is defined as the absence or critical impairment of one or more vital functions, requiring immediate medical evaluation.

### 2.2. Data Collection

Patients were eligible for inclusion if they were aged between 0 and 16 years at the time of presentation to the PED between 1 January 2021 and 1 July 2025, receiving an RC designation at triage. Patients with suspected child abuse, psychomotor agitation and those brought to the PED in out-of-hospital cardiorespiratory arrest with no measurable vital signs upon arrival were excluded. Patients with suspected child abuse were excluded because, although requiring urgent assessment, these cases are primarily driven by safeguarding needs rather than acute physiological deterioration and may not present with clinical or laboratory abnormalities or require emergency treatment. Their inclusion could therefore have confounded the identification of early markers of deterioration and adverse outcomes.

Clinical, instrumental, diagnostic, and therapeutic data were collected from the electronic medical records of patients admitted to the PED.

Data were collected from admission through discharge. During hospitalization, the following time intervals were considered: 0–48 h; 49–72 h; 73 h–7 days; and at the time of discharge.

### 2.3. Definitions and Parameters

Data on epidemiologic characteristics, PED presentation, initial clinical management, laboratory and instrumental investigations, early therapeutic interventions, and patient outcomes—including disposition to the SOU or pediatric ward, PICU admission, or death—were extracted from the hospital’s electronic medical record system.

Comorbidities included preexisting and chronic health conditions, defined as any medical condition expected to last at least 12 months (unless death occurs) and involving either multiple organ systems or a single organ system to a degree requiring specialty pediatric care and hospitalization at a tertiary care center [10]. Chronic therapy was defined as the ongoing use of disease-specific medications associated with the identified comorbidity.

Cardiovascular (capillary refill time and heart rate) and respiratory (respiratory rate and oxygen saturation) parameters were assessed, while neurological status was defined according to Pediatric Advanced Life Support guidelines based on Glasgow Coma Scale values. Deviations from age-specific normal values were evaluated using a composite continuous score (range: 0–2 per parameter, Supplementary Table S2), with higher scores indicating greater impairment. When one parameter within a domain was missing, the available value was applied to both parameters. The score was analyzed as a continuous variable without predefined severity cutoffs [4,11,12,13].

RC presentations were subsequently categorized into predefined diagnostic groups according to the primary underlying condition, including respiratory failure, hypovolemic shock, hemorrhage, cardiogenic shock, distributive shock due to sepsis, distributive shock due to anaphylaxis, neurological disorders, head trauma or intracranial hypertension, metabolic emergencies, poisoning, and surgical conditions.

Advanced life support (ALS) was operationally defined as the need for escalation of cardiovascular or respiratory support, including a rapid intravenous fluid bolus of 10 mL/kg for hemodynamic support, vasoactive/inotropic therapy, or invasive mechanical ventilation. Although these interventions differ in intensity, they were grouped into a composite ALS variable because they all represented escalation beyond routine supportive care and were considered markers of increased need for organ support relevant to PICU admission [14].

### 2.4. Statistical Analysis

Qualitative variables were reported as counts and percentages. Quantitative variables were assessed for normality using the Kolmogorov–Smirnov test and graphical methods; non-normally distributed variables were expressed as median (IQR) and normally distributed variables as mean ± SD. Univariable logistic regression was used to screen potential predictors, and variables with *p* < 0.20 were included in the multivariable models.

Independent variables included age, medically assisted transport, presence of chronic comorbidities, clinical score, and ALS interventions provided during the PED stay. Although ALS interventions occurred after presentation to the PED, they were included as independent variables because they represented early markers of clinical deterioration and escalation of care during the PED stay and were hypothesized to be associated with subsequent clinical outcomes. Dependent variables included ward admission, PICU admission, need for advanced vital support within 7 days (mechanical ventilation and/or hemodynamic support, including fluid boluses or inotropic agents), and hospitalization at 7 days. An ordinal logistic regression analysis was performed, with the same independent variables and level of care as the dependent variable. The proportional odds assumption was tested and met. Missing data were minimal and assumed to be missing completely at random (MCAR). Statistical significance was set at *p* < 0.05. Analyses were performed using R software, version 4.3.2 (R Foundation for Statistical Computing).

## 3. Results

During the 2021–2025 period, 254 RC cases were recorded (0.23% of 108.000 total PED visits), of which 153 met the study inclusion criteria. Four children were excluded due to out-of-hospital cardiorespiratory arrest with no measurable vital signs upon PED arrival, one due to psychomotor agitation, and 96 due to suspected child abuse.

The median age was 4.3 (IQR: 1.6–9.2) years. The largest age group was school-age children (6–12 years; 27.45%), followed by toddlers (1–2 years; 26.14%) and preschool-age children (3–5 years; 20.92%). The sex distribution was balanced, with a slight male predominance (52.94% vs. 47.06%) (Table 1). The majority of presentations occurred during daytime hours (8:00 am–8:00 pm; 60.78%) compared with nighttime (8:00 pm–8:00 am; 39.22%). Most patients arrived by medical transport (67.32%), whereas a minority arrived independently (32.68%) (Supplementary Table S2).

Overall, 56.21% of patients had at least one pre-existing chronic condition. The most common comorbidity was a neuropsychiatric condition (54.49%), followed by cardiovascular (12.79%), onco-hematological (11.63%), endocrine-metabolic (8.14%), and respiratory (8.14%) disorders, and less commonly, surgical-gastrointestinal (4.65%) and nephrological (1.16%) disorders. Overall, 57.52% of patients were not receiving any pharmacological treatment, 24.83% were on antiepileptic therapy and 5.88% were receiving cardiovascular medications.

### 3.1. Red Code Characteristics

Neurological alterations, particularly seizures, represented the leading cause of RCs (33.99%), followed by respiratory conditions (25.49%) and metabolic derangements (9.15%), the latter predominantly involving glycemic abnormalities. Heart failure (8.50%) and drug- or substance-related intoxications (7.19%) were also observed with notable frequencies.

Physiological parameters recorded at PED presentation showed variable degrees of compromise across clinical domains. Cardiovascular findings were generally mild: CRT was normal in most patients (90.51%), HR fell within age-adjusted percentiles in 39.19% (tachycardia in 94.4% of abnormal cases), and among cases with available arterial blood pressure measurements, 97.68% were within the normal range; blood pressure data were unavailable for 67 of 153 cases (43.79%). Respiratory involvement was common, with RR abnormalities observed in 52.00% of cases and respiratory support required in 46.40% of patients. Neurological impairment was also common, with nearly half of the cohort presenting with a GCS ≤ 12 (49.60%), while temperature abnormalities—mainly fever—were observed in approximately one quarter of cases.

### 3.2. Therapeutic Interventions

Respiratory support was required in 46.40% of patients: low-flow oxygen therapy was used in 26.14% of cases, Continuous Positive Airway Pressure (CPAP)/High-Flow Nasal Cannula (HFNC) in 6.53%, non-invasive ventilation (NIV) in 6.53%, and mechanical ventilation in 7.18%. Fluid bolus administration represented the main circulatory stabilization strategy (18.95%), whereas inotropic support was limited to the most severe cases (3.92%). Cardiopulmonary resuscitation (CPR) was performed in four children, and rhythm cardioversion (AED) in a single case.

### 3.3. Outcomes and Hospitalization

**Figure 1:**
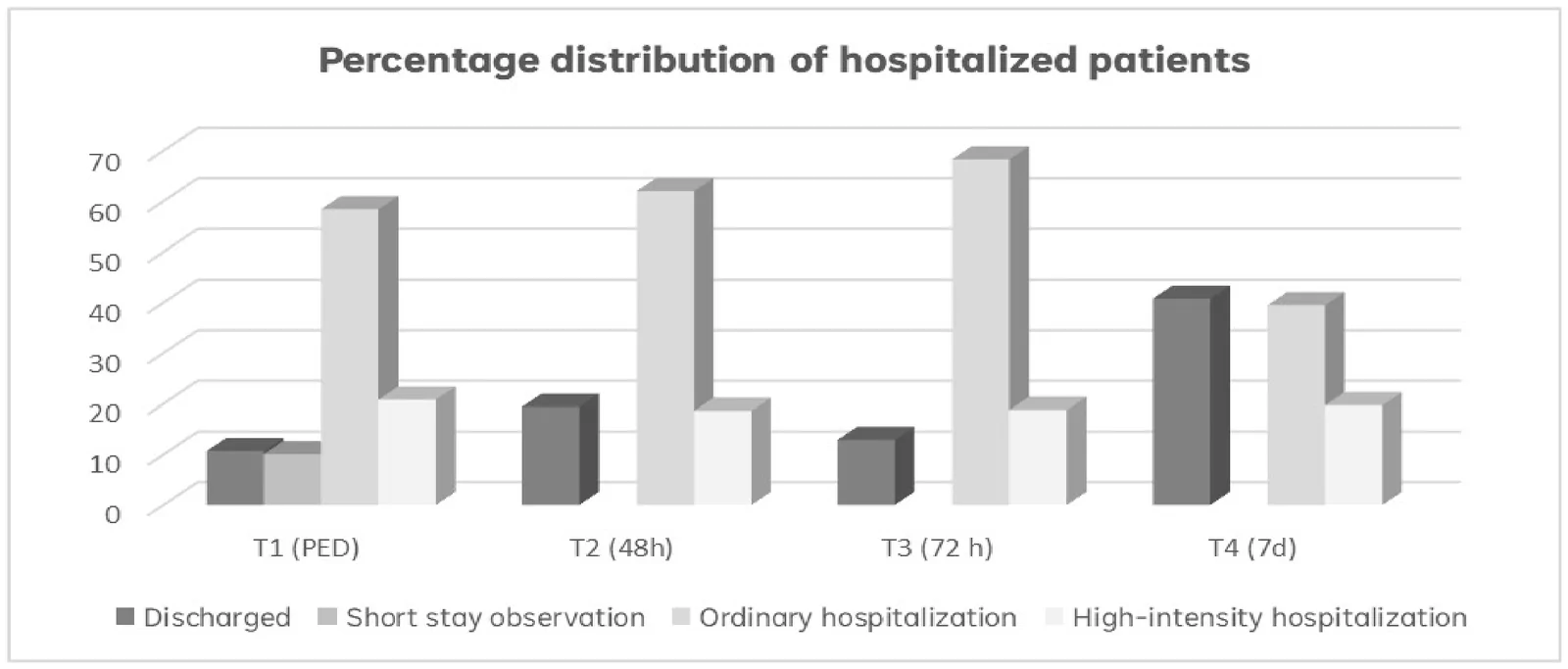
Level of care over time: ward and intensive care unit admissions at T1 (ED disposition), T2 (48 h), T3 (72 h), and T4 (7 days).

PED disposition showed that most children required hospitalization. Over half of the cohort was admitted to the pediatric ward (56.86%), while 20.26% required ICU admission. A smaller proportion was discharged directly (10.46%) or after short-stay observation (9.80%). Death in the PED occurred in 2.61% of cases. The median length of hospital stay was 5 (IQR: 2–9) days. After the first 48 h, 62.02% of the admitted children were in a standard ward, 18.60% still required ICU care and 19.38% were discharged; at 72 h, these proportions changed to 68.32%, 18.81%, and 12.87%, respectively. After one week, an additional 40.78% of hospitalized children had completed their stay (overall, 70.39% of RC patients had been discharged), while among the remaining inpatients, 33.33% were in the NICU/PICU (eight patients on advanced life support) and 66.67% in low-intensity care (Figure 1).

### 3.4. Deaths

In our cohort, excluding four out-of-hospital deaths, a total of 10 in-hospital deaths were recorded: four of them occurred in the PED, four within the first 72 h of hospitalization, and two within 10 days after admission. The sex distribution was balanced (five males and five females). The overall mortality rate among RC presentations was 6.53%; specifically, the mortality rate was 2.61% in the PED and 3.92% among hospitalized patients.

Advanced life support was activated in nine of 10 (90%) cases. Patients without pre-existing chronic conditions were generally younger (mean age: 26 months) compared with those with chronic comorbidities (mean age: 96 months). Among previously healthy children, the predominant cause of death was sudden cardiac arrest consistent with sudden infant death syndrome (SIDS), whereas among children with chronic diseases, respiratory failure was the leading cause of death (three cases). In four cases, inotropes, fluid boluses, and advanced ventilatory support were initiated in the PED; three involved infants aged ≤13 months, and all four died in the PED. Among the five children with chronic conditions, three were admitted to a general ward and died within 72 h while receiving non-invasive respiratory support (Table 2).

### 3.5. Prognostic Parameters in the PED

**Figure 2:**
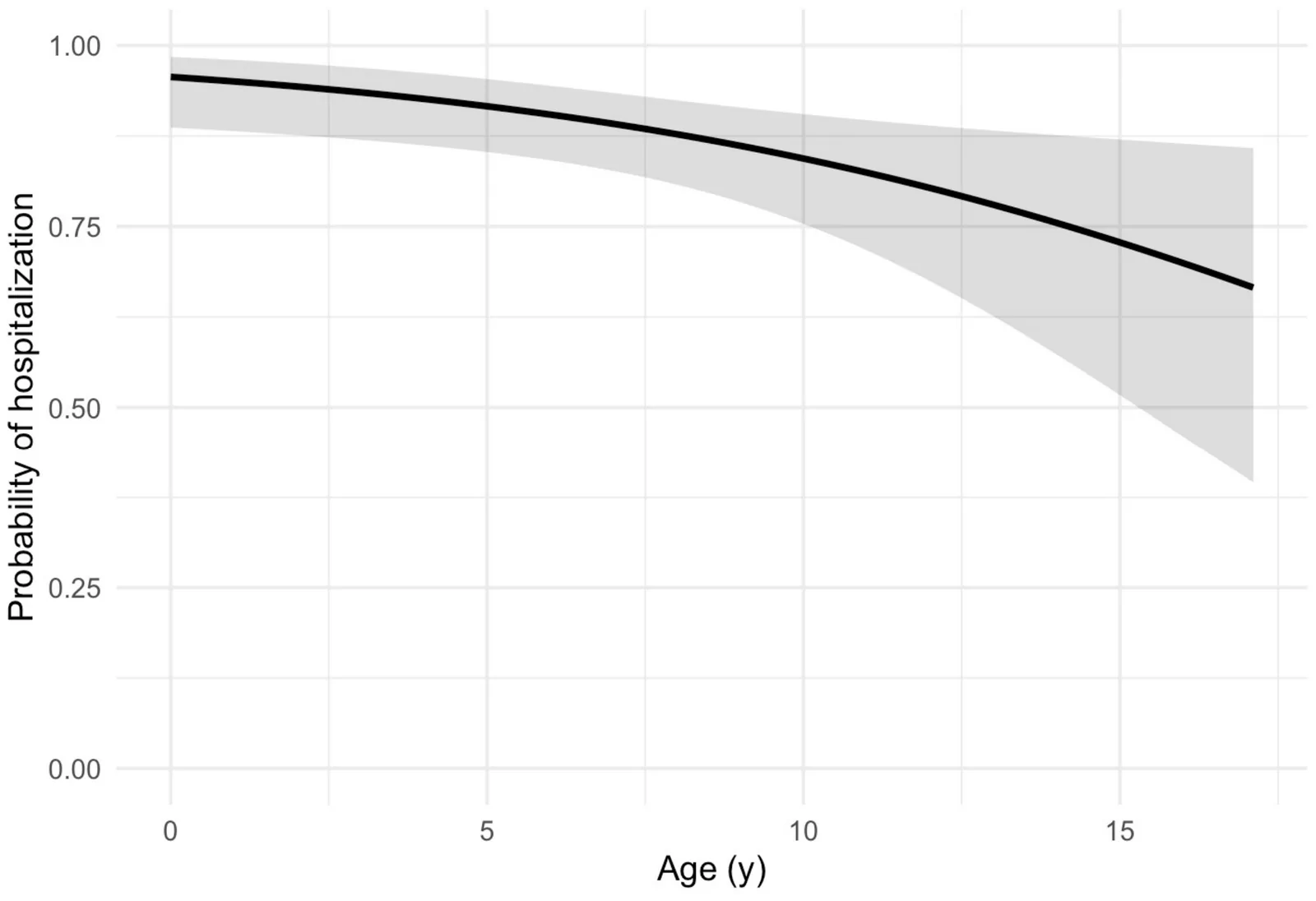
Predicted probability of hospitalization by age.

In the regression analyses, increasing age was associated with significantly lower odds of both hospital admission (OR: 0.876, 95% CI: 0.783–0.976; *p* = 0.017) and PICU admission (OR: 0.890, 95% CI: 0.793–0.983; *p* = 0.031) with risk reductions of 12.4% and 11.0% per additional year of age, respectively (Figure 2). Conversely, the need for advanced life support in the ED was independently associated with PICU admission (OR: 3.247, 95% CI: 1.345–7.997; *p* = 0.009). No statistically significant predictors were identified for the need for life support at 7 days and for discharge status or level of care at 7 days (Table 3).

## 4. Discussion

This study provides a detailed and comprehensive characterization of children presenting to our PED with RC triage that is consistent with previous reports. In our cohort, RCs accounted for 0.23% of PED visits, a proportion within the range described in the literature, although at the lower end. This finding may be explained by the organizational structure of our institution, which does not include a trauma center, as trauma-related conditions account for approximately 12–25% [1,3,5,15,16].

Consistent with previous investigations, we found that younger patients are more likely to be admitted than older patients. However, no association was observed between age and discharge within 7 days. We therefore hypothesize that the reduced functional reserve of children in the first years of life leads PED physicians to adopt a cautious approach with observation in a protected setting [5,17,18,19].

In our cohort, clinical severity—reflected by the need for advanced life support in the PED rather than by elevated clinical score—showed a stronger association with PICU admission. The small number of children still requiring vital support at 7 days precluded a meaningful statistical correlation with PED findings. Nevertheless, this observation suggests a generally favorable short-term recovery despite the initial severity of presentation [11,20,21,22].

Overall, our findings suggest that age and clinical severity may have different prognostic implications depending on the outcome considered. Younger age was associated with hospital and PICU admission, whereas neither age nor clinical severity showed a statistically significant association with outcomes assessed at 7 days. However, the lack of significant associations at 7 days should be interpreted with caution given the relatively small sample size and the limited number of events, which may have reduced the statistical power of the regression models to detect clinically relevant associations. This may be partly explained by the characteristics of our study population, which only included patients triaged with the highest-acuity code, resulting in a relatively homogeneous and clinically compromised group. Despite sharing a similar degree of clinical severity, these patients presented with heterogeneous underlying diagnoses, representing a potential source of confounding that should be taken into account. Such restricted variability in severity, together with diagnostic heterogeneity, may have limited our ability to detect consistent associations, particularly for outcomes assessed beyond the initial acute phase, compared with studies on unselected PED populations across the full spectrum of triage acuity. Further prospective studies with larger cohorts and a broader spectrum of triage acuity are therefore needed to confirm these findings and to identify reliable prognostic markers for pediatric patients presenting with high-acuity emergencies in the PED.

Notably, nearly half of the children in our cohort had at least one pre-existing comorbidity, highlighting the substantial burden of underlying conditions among critically ill patients presenting to the PED. This finding may suggest a particularly vulnerable patient population in a pediatric setting.

In recent years, the prevalence of chronic conditions has steadily increased, likely driven by advances in medical care. In our cohort, neurological diseases represented the largest subgroup, followed by onco-hematological and cardiac conditions, reflecting our center’s role as an interregional referral center for these patients [23,24].

Children with chronic conditions accounted for 26% of hospital inpatient days (their hospital stays were three times longer than those of children without chronic conditions, with a threefold increased risk of ICU admission) and 40% of hospital expenditures, primarily due to increased need for specialist evaluations and invasive procedures such as gastrostomy, tracheostomy, and cerebrospinal fluid shunt placement [23,25].

Furthermore, in our cohort, 25% of children with chronic conditions were followed by pediatric palliative care services. This finding highlights the need for structured follow-up and for strengthening tertiary prevention strategies within the community care network to reduce potentially avoidable PED admissions in the coming years.

The most frequently performed life-saving interventions were related to respiratory support and advanced airway management was required in approximately one in ten critically ill patients. By contrast, circulatory instability appeared to be less common and rapid fluid bolus administration represented the main strategy for hemodynamic stabilization, whereas cardiopulmonary resuscitation interventions and electrical cardioversion were rarely required.

Technical competence is closely linked to procedural exposure. However, pediatric patients generally present with lower acuity than adults, and only 1–5% of cases require life-saving interventions—approximately one-third of the intubation rate and less than one-quarter of the CPR rate reported in adults [1,17]. Consequently, opportunities to maintain procedural competence in pediatric emergency care are limited [3,16,26]. In this context, simulation-based training has been proposed as a valuable strategy to maintain procedural readiness [15]. Several studies have demonstrated improvements in technical skills, anxiety management, and the identification of latent system threats, such as equipment shortages or ambiguities in local protocols [27].

From the PED, most RC patients were transferred to standard wards, while approximately one-fifth required admission to the ICU. The proportion of patients admitted directly to the PICU from the ED was consistent with previous reports [1,3,5,15].

The mortality rate among patients presenting with RC triage in our center was 6.53%, which is within the range reported in the literature (0.9% to 7.2%) [1,3,5,15].

In the general pediatric population, mortality is estimated to be 23–29 deaths per 100,000 children, markedly higher than the mortality following a PED visit, which has been estimated to be 2–7 deaths per 100,000 children [3,28,29,30]. The PED is therefore not a setting in which pediatric deaths occur more frequently; most pediatric deaths occur during inpatient hospitalization or in high-acuity hospital units [6]. Deaths occurring in the ED are more commonly related to sudden cardiac death (26%), infections (24%), and surgical conditions (16%) [1,28,30].

## 5. Study Limitations

The limitations of the study include the retrospective design, which carries a risk of information bias and misclassification related to incomplete data, heterogeneity of documentation practices, and variability in data quality. The single-center design may reduce the generalizability of the results to other clinical settings with different case mixes, resources, and emergency care pathways, and may have introduced a selection of more severe cases. Although a multivariable approach was planned, the limited number of outcome events constrained the number of covariates that could be reliably included in the models as it could have led to a residual risk of overfitting, reduced statistical power to detect independent associations, and potential residual confounding.

## 6. Conclusions

This study provides a portrait of the pediatric population presenting with RC that is consistent with international literature while also reflecting the unique characteristics of the local healthcare system. Our cohort demonstrates a high prevalence of chronic comorbidities, confirming the growing relevance of these conditions in PEDs and in hospital resource allocation, with particular attention to the implementation of pediatric palliative care services. Life-saving interventions were rarely required, underscoring the need for targeted strategies to maintain and enhance medical competencies. Younger age was associated with an increased risk of hospitalization, likely reflecting a cautious approach by physicians. In addition, measurement of clinical parameters in the ED cannot replace a comprehensive medical assessment of the underlying condition.

## Figures and Tables

**Table 1:** Clinical and demographic characteristics of the study population, including the number of diagnostic and therapeutic interventions in the emergency department and patient disposition at ED discharge. MRI: Magnetic Resonance Imaging; CT: Computed Tomography; ECG: electrocardiogram; EEG: electroencephalography; CPR: cardiopulmonary resuscitation; AED: Automated External Defibrillator; CPAP: Continuous Positive Airway Pressure; HFNC: High-Flow Nasal Cannula; PED: pediatric emergency department.

Variable	N (153)	%
Neonate (0–30 days)	5	3.27
Infant (1 month–11 months)	18	11.76
Toddler (1–2 years)	40	26.14
Pre-school (3–5 years)	32	20.92
School (6–12 years)	42	27.45
Adolescent (13–16 years)	16	10.46
Female/male	72/81	47.06/52.94
Pre-existing chronic condition	86	56.21
Chronic therapies	65	42.48
Drugs with hemodynamic impact	9	5.88
Drugs with respiratory impact	5	3.27
Immunosuppressants/chemotherapy	3	1.96
Neurological drugs	38	24.83
Hormonal–metabolic drugs	5	3.27
Nutritional support	5	3.27
Diagnosis		
Respiratory failure	39	25.49
Hypovolemic shock	4	2.61
Hemorrhage	3	1.96
Cardiogenic shock	13	8.5
Distributive shock (sepsis/anaphylaxis)	3/1	2.61
Neurological pathology	52	33.99
Head trauma/intracranial hypertension	8	5.23
Metabolic emergency	14	9.15
Poisoning	11	7.19
Surgical pathology	5	3.27
Diagnostic Interventions		
Ultrasound	12	7.84
Radiography	61	39.86
MRI/CT	31	20.26
ECG	35	22.87
EEG	35	22.87
Operating room endoscopy (pre-admission)	7	4.57
Etiological Therapy	136	88.89
Bronchodilators/anti-inflammatory	24	15.68
Transfusions	4	2.61
Anti-arrhythmic/cardiac drug	3/2	2.27
Antibiotic	12	7.84
Antiepileptic drug	45	29.41
Glucose bolus	5	3.27
Fluid therapy	7	4.58
Insulin	6	3.92
Detoxifier	8	5.23
CPR/AED	4/1	3.26
Minor surgical procedure	6	3.92
Major surgical procedure	9	5.88
Cardiovascular support	35	22.87
Inotropes	6	3.92
Fluid boluses	29	18.95
Respiratory support	71	46.4
Oxygen therapy (low-flow)	40	26.14
CPAP/HFNC	10	6.53
Non-invasive ventilation	10	6.53
Mechanical ventilation	11	7.18
Advanced life support team activation	60	39.22
PED outcome		
Discharged	16	10.46
Short-stay observation	15	9.8
Ordinary hospitalization	87	56.86
Intensive care unit	31	20.26
Death	4	2.61
Alive/Dead	143/10	93.46/6.54

**Table 2:** Description of clinical characteristics observed in the emergency department among patients who died during the study period. M: male; F: female; ED: emergency department; PICU: pediatric intensive care unit.

Patient/ Sex	Age (Months)	Comorbidity	Emergency	Inotropes	Fluids	Respiratory Support	Anesthetist	Outcome After Leaving ED
1/M	0.6	None	SIDS	≥2	Bolus	Invasive ventilation	Yes	Death
2/M	13.2	None	SIDS	1	Bolus	Invasive ventilation	Yes	Death
3/F	5.4	None	SIDS	≥2	Bolus	Invasive ventilation	Yes	Death
4/F	79.9	None	Sepsis	0	Maintenance fluid	Non-invasive vent.	Yes	PICU
5/M	30.2	None	Metabolic neuropathy	0	Maintenance fluid	Self-ventilating	Yes	PICU
6/M	81.1	Respiratory disorder	Respiratory failure	0	0	Invasive ventilation	Yes	Standard ward
7/F	147.1	Neuropsychiatric	Respiratory failure	0	Bolus	Non-invasive vent.	Yes	PICU
8/F	59.1	Onco-hematological	Intracranial hypertension	0	0	Self- ventilating	No	Standard ward
9/M	33.3	Neuropsychiatric	Respiratory failure	0	0	Low-flow oxygen	Yes	Standard ward
10/F	162.3	Cardiovascular	Heart failure	≥2	Bolus	Invasive ventilation	Yes	Death

**Table 3:** Univariable and multivariable logistic regression analyses evaluating factors associated with hospital admission, PICU admission, advanced life support (ALS) at 7 days, and discharge at 7 days. Ordinal logistic regression analysis was performed for level of care. Odds ratios (ORs), 95% confidence intervals (CIs), and *p* values are reported.

Covariate	Univariable Logistic Regression	Multivariable Logistic Regression					
Hospital admission:		OR	95% CI	*p*	OR	95% CI	*p*
Age (y)		0.869	0.776–0.968	**0.012**	0.876	0.783–0.976	**0.017**
Medical transport		0.888	0.266–2.605	0.836	-	-	-
Comorbidity		0.825	0.267–2.355	0.724	-	-	-
Advanced life support		1.433	0.468–5.360	0.553	-	-	-
Vital parameters score		1.287	1.006–1.710	**0.058**	1.285	0.983–1.656	0.089
PICU admission							
Age (y)		0.912	0.822–1.001	**0.064**	0.890	0.793–0.983	**0.031**
Medical transport		1.965	0.815–5.280	**0.151**	1.984	0.785–5.581	0.166
Comorbidity		0.832	0.375–1.868	0.652	-	-	-
Advanced life support		2.993	1.325–6.833	**0.008**	3.247	1.345–7.997	**0.009**
Vital parameters score		1.158	0.977–1.377	**0.091**	1.094	0.905–1.324	0.353
ALS at 7 days							
Age (y)		0.950	0.765–1.133	0.598	-	-	-
Medical transport		3.250	0.501–64.044	0.292	-	-	-
Comorbidity		1.555	0.309–11.570	0.616	-	-	-
Advanced life support		0.694	0.128–3.215	0.646	-	-	-
Vital parameters score		1.127	0.845–1.525	0.415	-	-	-
Discharge at 7 days							
Age (y)		1.001	0.902–1.113	0.980	-	-	-
Medical transport		0.770	0.267–2.112	0.617	-	-	-
Comorbidity		1.647	0.642–4.259	0.298	-	-	-
Advanced life support		1.454	0.571–3.803	0.436	-	-	-
Vital parameters score		1.017	0.844–1.231	0.853	-	-	-
		Ordinal logistic regression					
Level of care at 7 days							
Age (y)		0.953	0.858–1.055	0.355	-	-	-
Medical transport		0.854	0.327–2.234	0.747	-	-	-
Comorbidity		1.632	0.618–4.397	0.325	-	-	-
Advanced life support		1.393	0.557–3.511	0.478	-	-	-
Vital parameters score		0.983	0.809–1.192	0.862	-	-	-

## Data Availability

The data presented in this study are available from the corresponding author upon reasonable request. The data are not publicly available because they contain information that could compromise participant privacy and are subject to institutional data protection policies.

## Supplementary Materials

The following supporting information can be downloaded at: https://www.mdpi.com/article/10.3390/children13091151/s1, Table S1. Pediatric emergency department triage system according to the general guidelines of the Emilia-Romagna Region (Italy). Table S2. Other emergency department findings and vital signs, categorized according to the degree of deviation from age-specific normal values.
