# Supplementary material for: From Triage to Outcome: How ED Parameters and Comorbidities Shape Pediatric Critical Illness Prognosis
Source: Children (Basel). 2026 Aug 27;13(9):1151. doi: 10.3390/children13091151 (PMC13605866; doi:10.3390/children13091151)
Supplement: Supplementary file 1 [file children-13-01151-s001.zip › children-4468757-supplementary.pdf]

Supplementary

Table S1. Pediatric emergency department triage system according to the general guidelines of the Emilia-Romagna Region (Italy).

Priority Code	Urgency Level	Detailed Definition	Maximum Waiting Time for Initial Assessment
**1 RED**	EMERGENCY	Absence or critical impairment of one or more vital functions.	Immediate
**2 ORANGE**	NON-DEFERRABLE URGENCY	Clinically stable condition with a high risk of rapid deterioration.	15 min
**3 BLUE**	DEFERRABLE URGENCY	Stable condition with a low risk of deterioration but associated with significant discomfort or impact on the general condition, usually requiring complex diagnostic or therapeutic interventions.	60 min
**4 GREEN**	MINOR URGENCY	Stable condition without risk of deterioration and without significant discomfort or impact on the general condition; usually does not require complex interventions.	120 min
**5 WHITE**	NON-URGENT	Non-urgent medical issue.	240 min

Table S2. Other emergency department findings and vital signs, categorized according to the degree of deviation from age-specific normal values.

Variable	N (153)	%
Mode of arrival		
Independently	50	32.68
Medical transport	103	67.32
Time of arrival		
Daytime (8-20)	93	60.78
Nighttime (20-8)	60	39.22
Vital parameters		
Heart rate (missing: 5)		
0 (10-90°p)	58	39.19
1 (2-10°p or 90-98°p)	26	17.57
2 (≤1 or ≥99°p)	64	43.24
Blood pressure (missing: 67)		
0	84	97.68
1	1	1.16
2	1	1.16

Blood pressure scores in this study were assigned according to the Phoenix Pediatric Sepsis Score classification, using age-specific thresholds for mean arterial pressure.

Temperature (missing: 2)		
0: 35.1-37.7		
1:37.8-39.4 °C or 32-35 °C	111	73.51
2: ≥ 39.5 °C or < 32°C	33	21.85
	7	4.64
Refill time (missing: 16)		
0: <2 sec		
1: 2-3 sec	124	90.51
2: ≥ 4 sec	9	6.57
	4	2.92
Respiratory rate (missing: 53)		
0 (10-90°p)		
1 (2-10°p or 90-98°p)	48	48.0
2 (≤1 or ≥99°p)	19	19.0
	33	33.0
SpO2 and respiratory support (missing: 2)		
0: >95 Self Ventilating (SV)		
1: >95 with Respiratory Support (RS)or 90-94 SV	81	53.6
2: < 95 with RS or <90 SV	32	21.2
	38	25.2
GCS		
0: 13-15		
1: 9-12	77	50.4
2: 3-8	38	24.8
	38	24.8
